# 3-styrylcoumarin scaffold-based derivatives as a new approach for leishmaniasis intervention: biological and molecular modeling studies

**DOI:** 10.1007/s12639-023-01639-x

**Published:** 2024-02-01

**Authors:** Andrés F. Yepes, Sara M. Robledo, Jorge Quintero-Saumeth, Wilson Cardona-Galeano

**Affiliations:** 1 Chemistry of Colombian Plants, Faculty of Exact and Natural Sciences, Institute of Chemistry, University of Antioquia-UdeA, Calle 70 No. 52-21, A.A 1226, Medellín, Colombia; 2 Faculty of Medicine, PECET-Medical Research Institute, University of Antioquia-UdeA, Calle 70 No. 52-21, A.A 1226, Medellín, Colombia

**Keywords:** 3-styrylcoumarins, Leishmaniasis, Docking studies, Molecular modeling studies, In-silico pharmacokinetic evaluation

## Abstract

Seven 3-styrylcoumarins were tested for antileishmanial activity against *Leishmania* (Viannia) *panamensis* amastigotes. Cytotoxic activity was also evaluated against mammalian U-937 cells. The 3-methoxy-4-hydroxy coumarin derivative **6** was the most active with an IC_50_ of 40.5 µM, and did not reveal any conspicuous toxicity toward mammalian U-937 cells. Therefore, it may have potential to be considered as candidate for antileishmanial drug development. Further, among several druggable Leishmania targets, molecular docking studies revealed that compound **6** had docking preference by the *N*-myristoyltransferase (*Lp*-NMT) of *Leishmania panamensis*, showing a higher docking score of − 10.1 kcal mol^−1^ than positive controls and making this protein as a presumably druggable target for this compound. On the other hand, molecular dynamics simulations affirm the docking hypothesis, showing a conformational stability of the **6**/*Lp*-NMT complex throughout 100 ns simulation. Moreover, the molecular mechanics/Poisson–Boltzmann surface area method also support the docking findings, revealing a total free energy of binding of − 47.26 ± 0.08 kcal mol^−1^, and identifying through energy decomposition analysis that those key aminoacids are contributing strongly to ligand binding. Finally, an optimal pharmacokinetic profile was also estimated for **6**. Altogether, coumarin** 6** could be addressed as starting point for further pharmacological studies concerning the therapeutic leishmaniasis intervention.

**Graphical abstract:**

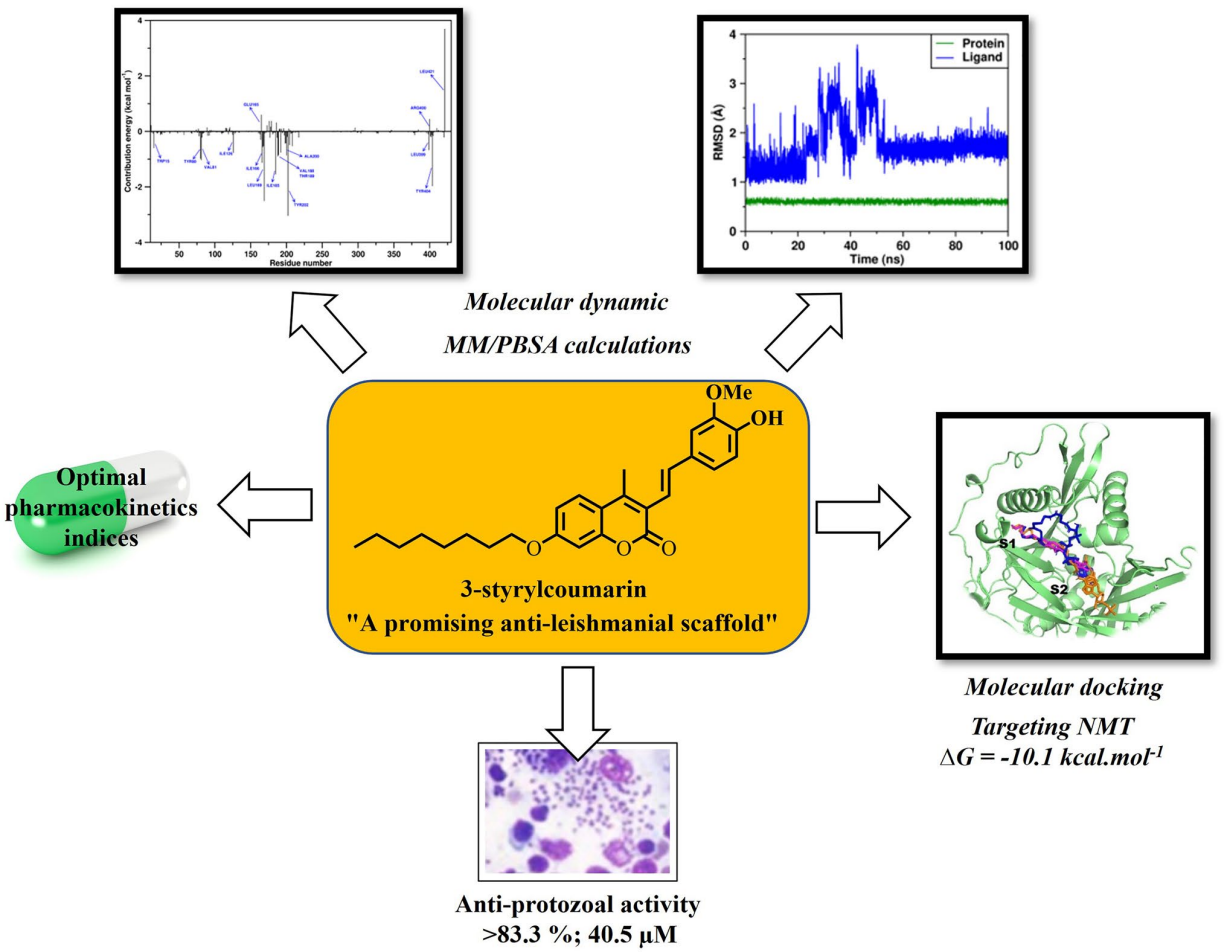

## Introduction

Protozoal diseases (PD) are a diverse group of diseases, which are the cause of significant mortality in various developing countries of tropical and subtropical regions. PDs include, among others, leishmaniasis which is caused by the parasitic protozoan of *Leishmania* species (WHO 2022a, 2022b). Current chemotherapies are still based on old drugs such as pentavalent antimonials (meglumine antimoniate and sodium stibogluconate), pentamidine isethionate and amphotericin B to treat cutaneous leishmaniasis (WHO 2023); Unfortunately, these therapeutic treatments have several toxic effects on the patients that are associated with high doses and length of therapeutic schemes. Moreover, they are no longer as effective as before due to the emergence of drug resistance in the parasite, which complicates the control of these diseases (Pacheco et al. 2023; eBioMedicine 2023; Chatelain et al. 2011; Den Boer et al. 2011); Keenan and Chaplin 2015).

**Fig. 1:**
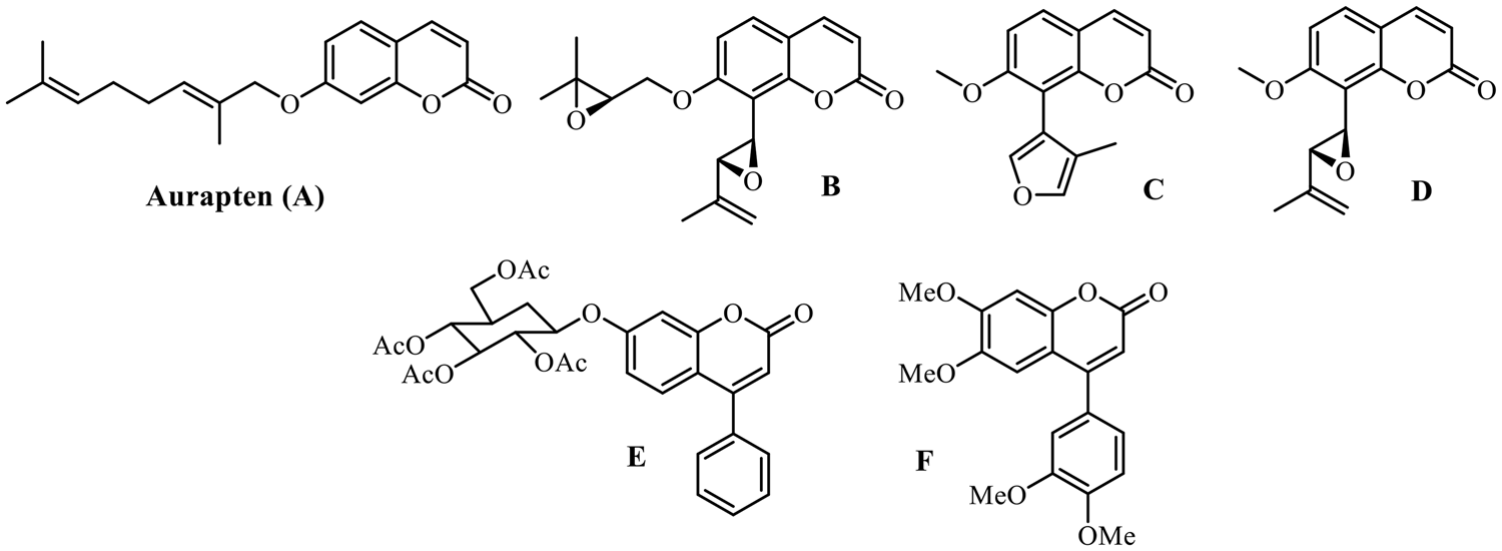
Coumarins with antileishmanial activity: Aurapten (**A**), coumarins from *Galipea panamensis* (**B**–**D**), 7-glucocoumarin (**E**), 4-arylcoumarin (**F**)

Coumarins are an important class of compounds having versatile biological activities (Flores et al. 2023; Annunziata et al. 2020). Aurapten (**A**), a 7-geranyloxycoumarin, was extracted from the Rutaceae species *Esenbeckia febrifuga* (Santos et al. 2023). This compound shows significant growth inhibition with a 50% inhibitory concentration (IC_50_) of 30 µM against *L. major* (Napolitano et al. 2004). Three coumarins isolated from the leaves of *Galipea panamensis* (**B–D**) were tested against axenic amastigote forms of *L. panamensis* and displayed 50% effective concentrations (EC_50_) of 9.9, 10.5, and 14.1 µg/mL, respectively (Arango et al. 2010). Coumarin **E**, presented IC_50_ values of 10.03 and 34.93 µM against promastigote and amastigote forms of *L. amazonensis*, respectively (Rosa et al. 2017). In addition, 4-arylcoumarin (**F**) was found to strongly inhibit the protozoan parasites of *L. donovani.* This compound exhibited an IC_50_ value of 1.1 µM on intracellular amastigotes with a selectivity index (SI) of 265, almost twice that shown by amphotericin B (SI = 140) (Pierson et al. 2010) (Fig. 1).

In this work, a series of 3-styrylcoumarins were evaluated in vitro to test their antileishmanial activity against intracellular amastigotes of *L. panamensis*. Cytotoxicity of these compounds was tested on U-937. Moreover, molecular modeling studies were conducted.

## Materials and methods

### Biological

#### In vitro cytotoxicity

Cytotoxic activity of the studied compounds was assessed in the human promonocytic U-937 cells (ATCC CRL-1593.2™) based on viability evaluated by the MTT (3-(4,5-dimethylthiazol-2-yl)-2,5-diphenyltetrazolium bromide) assay as described elsewhere (Taylor et al. 2011). Briefly, U-937 cells grown in tissue flasks were harvested and washed with phosphate buffered saline (PBS) by centrifugation. Cells were counted and adjusted at 1 × 10^6^ cells/mL of complete culture medium (RPMI-1640 supplemented with 10% Fetal Bovine Serum-FBS and 1% of antibiotics (100 ug/mL penicillin and 0.1 mg/mL streptomycin). One hundred µL of cell suspension were dispensed into each well of a 96-well cell-culture plate and then 100 µL of two-fold serial dilutions of the compounds (starting at 200 µg/mL) in complete RPMI 1640 medium were added. Plates were incubated at 37 °C, under 5% CO_2_ atmosphere during 72 h. Then, 10 µL of MTT solution (0.5 mg/mL) were added into each well, and plates were then incubated at 37 °C for 3 h. The formazan crystals were dissolved by adding 100 µL/well of dimethyl sulfoxide and 30 min incubation. Cell viability was determined according to the intensity of color (absorbance) registered as optical densities (O.D) obtained at 570 nm in a Varioskan™ Flash Multimode Reader-ThermoScientific, USA spectrophotometer. Cells cultured in absence of compounds were used as control of viability (negative control), while doxorrubicin was used as control for cytotoxic drugs. Non-specific absorbance was corrected by subtracting the O.D of the blank. Assays were conducted in two independent runs with three replicas per each concentration tested.

#### In vitro antileishmanial activity

The activity of compounds was evaluated on intracellular amastigotes of *L.* (V) *panamensis* transfected with the green fluorescent protein gene (MHOM/CO/87/UA140-EGFP) (Pulido et al. 2012). The effect of each compound was determined according to the inhibition of the infection evidenced by both, decrease of the infected cells and decrease of intracellular parasite amount. Briefly, U-937 human cells at a concentration of 3 × 10^5^ cells/mL in RPMI 1640 containing 0.1 μg/mL of phorbol-12-myristate-13-acetate (PMA) were dispensed into each one of a 24-well cell culture plate and then infected with 5 day-old promastigotes in a 15:1 parasites per cell ratio. Plates were incubated at 34 °C, 5% CO_2_ during 3 h and cells were washed two times with PBS to eliminate non internalized parasites. One mL of fresh complete RPMI 1640 medium supplemented with 10% FBS and 1% antibiotics was added into each well, cells were incubated again to guarantee multiplication of intracellular parasites. After 24 h of infection, culture medium was replaced by fresh culture medium containing each compound at 50–6.12 and 1.56 μg/mL and plates were incubated at 37 °C, 5% CO_2_. After 72 h, inhibition of the infection was determined. For this, cells were removed from the bottom plate with a trypsin/EDTA (250 mg) solution; recovered cells were centrifuged at 1100 rpm during 10 min at 4 °C, the supernatant was discarded and cells were washed with 1 mL of cold PBS and centrifuged at 1100 rpm during 10 min at 4 °C. The supernatant was discarded and cells were suspended in 500 μL of PBS and analyzed by flow cytometry (FC 500MPL, Cytomics, Brea, CA, US). All determinations for each compound including standard drugs were carried out by triplicate, in two independent experiments. Activity of the tested compounds was carried out in parallel with infection progress in culture medium alone and in culture medium with amphotericin B as antileishmanial drugs (positive controls).

### Data analysis

Cytotoxicity was determined according to cell growth (viability) and mortality percentages obtained for each isolated experiment (compounds, doxorubicin and culture medium). Results were expressed as 50 lethal concentrations (LC_50_), corresponding to the concentration necessary to eliminate 50% of cells, calculated by Probit analysis (Finney 1978). Percentage of viability was calculated by Eq. 1, where the optical density (O.D) of control corresponds to 100% of viability (cell growth).

1 $$\% {\text{ Mortality }} = { 1} - \left[ {\left( {{\text{O}}.{\text{D Exposed cells}}} \right)/\left( {{\text{O}}.{\text{D Control cells}}} \right) \, \times { 1}00} \right]$$

Antileishmanial activity was determined according to percentage of infected cells and parasite amount obtained for each experimental condition by flow cytometer. The percentage of infected cells was determined as the number of positive events by green fluorescence (parasites) in y-axis and Forward Scatte in x-axis. Then, the parasitic load in those infected cells was determined by a histogram of the mean fluorescence intensity (MFI) of those fluorescent parasites (Pulido et al. 2012).

Parasitemia inhibition was calculated by Eq. 2, where the MFI of control corresponds to 100% of parasitemia. In turn, inhibition percentage corresponds to 100% Parasitemia. Results of antileishmanial activity were expressed as 50% effective concentrations (EC_50_) determined by the Probit method (Finney 1978):

2 $$\% {\text{ inhibition }} = { 1}{-}\left[ { \, \left( {\text{MFI Exposed parasites}} \right)/\left( {\text{MFI Control parasites}} \right) \, \times { 1}00} \right]$$

## Computational methods

### Protein structure and setup

The potential mechanism of action of the promising coumarin **6** (Table 2) was explored against the most important druggable targets in *Leishmania* therapy. The *Leishmania* protein targets examined were Adenine phosphoribosyltransferase (APRT), Trypanothione synthetase (TryS), N-myristoyltransferase (NMT), Trypanothione reductase (TryR), Pteridine reductase 1 (PTR1), Tryparedoxin peroxidase (TXNPx), Glyceraldehyde-3-Phosphate Dehydrogenase (GAPDH), Arginase (ARG), Oligopeptidase B (OPB), Dihydroorotate dehydrogenase (DHODH). The experimental crystal structure for *L. panamensis* of these ten proteins is not available in the Protein Data Bank (PDB), hence, their 3D-structures were modelled. Thus, for 3D-structure of model prediction, the amino acid sequence of the ten enzymes for *L. panamensis* were obtained from the UniProt database (UniProtKB 2018). For template identification, the enzyme amino acid sequences in FASTA format were submitted to the SWISS-MODEL server (Schwede et al. 2003; Arnold et al. 2006) by searching against Protein Data Bank (PDB) proteins to develop a model with sufficient sequence coverage and sequence identity (more than 75%). The sequence which shows high homology to the target sequence was chosen as template (based on the Global Model Quality Estimation (GMQE) and RMSD values). PROCHECK (Laskowski et al. 1993) was used to check the structural rationality of the modelled 3D structures for selected enzymes constructed via SWISS-MODEL, and VERIFIED-3D (Eisenberg et al. 1997; Bowie et al. 1991) was used to determine the compatibility of each built 3D-model with its amino acid sequence. The alignment of the ten proteins models and their templates structures was carried out using The PyMOL Molecular Graphics System 2.0 program. A structural superposition was carried out to determinate the Root Mean Square Deviation (RMSD) between the positioning of the carbon atoms of both the template and the built 3D-model that is obtained from the alignment. For a good alignment, low RMSD values should be obtained (less than 1 Å). Finally, for docking calculations, the structures of the selected proteins were parameterized using AutoDock Tools (Morris et al. 2009). Gasteiger partial charges were calculated and polar hydrogens to facilitate the formation of hydrogen bonds were added.

### Ligand dataset preparation and optimization

Ligand used in this study is the most active coumarin **6** and two well-known modulators of the enzyme *N*-myristoyltransferase (NMT): a potent non-hydrolysable inhibitor (*S*-(2-oxo)pentadecyl-CoA) and Myristoyl-CoA as an essential cofactor for NMT activity. The structures of the ligands were parameterized using AutodockTools to add full hydrogens to the ligands, to assign rotatable bonds, to compute Gasteiger charges and saving the resulting structure in the required format for further use with AutoDock. All possible flexible torsions of the ligand molecules were defined using AUTOTORS in AutoDockTools (Morris et al. 2009, 1998) to promote the calculated binding energy with those selected Leishmania protein targets.

### Docking, molecular dynamics (MD) and MM/PBSA protocols

Our docking protocol was carried out using AutoDock Vina and default procedures to dock a flexible ligand to a rigid protein. Coumarin **6** was docked with ten modeled proteins of the *L. panamensis* that have been previously identified as druggable target proteins. Then, docking simulation was carried out on the reported catalytic domain where the enzyme residues are in close proximity to the known inhibitors or essentials cofactors, as *S*-(2-oxo)pentadecyl-Coa or Myristoyl-CoA in complex with *N*-myristoyltransferase (NMT). Once a potential binding site was identified, the active coumarins were docked to these enzymes-sites aiming to determine the most probable and the most energetically favorable binding conformations. To accomplish that, rigorous docking simulations involving a grid box to the identified binding site, Autodock Vina 1.1.2 (Trott et al. 2009) was used. The exhaustiveness was 20 for each protein-compound pair. The active site was surrounded by a grid box of 40 × 44 × 48 Å with a grid spacing of 1 Å. Affinity scores (in kcal/mol) given by AutoDock Vina for all ligands were obtained and ranked based on the free energy binding theory (more negative value means larger binding affinity). The resulting structures and the binding docking poses were graphically inspected to check the interactions using the DS Visualizer 2.5 or The PyMOL Molecular Graphics System 2.0 programs. For MD simulations (100 ns), MM-PBSA studies, and theoretical calculations of the drug-likeness parameters, the protocols and methodology are described in detail in a recent publication (Cardona et al. 2022).

## Results and discussion

### Chemistry

**Fig. 2:**
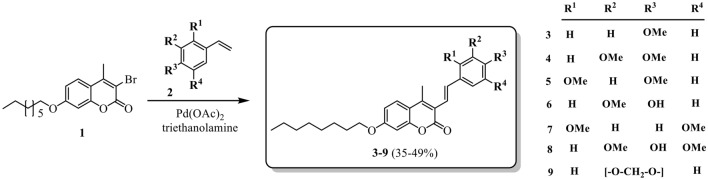
Synthetic pathway to 3-styrylcoumarins

The synthesis and characterization of the compounds under study has already been published (Herrera et al. 2018). 3-bromo-4-methyl-7-(octyloxy)-2*H*-chromen-2-one **1**, which was obtained from 7-hydroxy-4-methyl-2*H*-chromen-2-one through the reaction of Williamson's etherification with 1-bromooctane followed by bromination with NBS, and subjected to cross-coupling with various styrenes **2** under palladium catalysis (Heck reaction) leading to the formation of 3-styrylcoumarins (**3-9**) (Fig. 2).

### Biological evaluation

#### Assays

All compounds were subjected to in vitro evaluation of their cytotoxicity and antileishmanial activity against U-937 human macrophages and intracellular amastigotes of *L.* (V) *panamensis,* respectively. The results are summarized in Table 1.

**Table 1:** In vitro anti-leishmanial and cytotoxicity activity of 3-styrylcoumarins

Compound	Inhibition intracellular amastigotes growth (%)	Anti-leishmanial activity on intracellular amastigotes EC_50_ (µg/ml, µM)^a^	Cytotoxicity on U-937 cells LC_50_ (µg/ml, µM)^b^	SI
**3**	0.0	ND^e^	> 200.0; > 549	–
**4**	32.5 ± 0.8	ND^e^	26.0 ± 4.8; 66.0 ± 12.2	–
**5**	3.7 ± 0.8	NP^e^	> 200.0; > 507	–
**6**	**83.5 ± 5.0**	**15.4 ± 2.4; 40.5 ± 6.3**	19.7 ± 1.0; 51.8 ± 2.6	1.2
**7**	7.1 ± 1.5	ND^e^	> 200.0; > 507	–
**8**	0.0^c^	ND^e^	1.0 ± 0.1; 2.4 ± 0.2	–
**9**	0.0	ND^e^	> 200.0; > 529	–
**Meglumine antimoniate**^f^	79.4 ± 2.1^ g^	9.4 ± 2.1; 25.7 ± 5.7	416.4 ± 66.6; 1137.8 ± 182.0	44.3
**Amphotericin B**	62.7 ± 1.4^d^	0.05 ± 0.01; 0.05 ± 0.01	36.1 ± 7.5; 39.1 ± 8.1	722

Data represent, mean value + / − standard deviation

^a^EC_50_: effective concentration 50

^b^LC_50_: lethal concentration 50

^c^Dose used 1.0 µg/ml. It is not possible to increase the concentration due to its high toxicity

^d^Dose used 0.05 µg/ml

^e^NP, Not determined, because the % inhibition at 20 µg/ml was less than 50%; SI: selectivity index = LC_50_/EC_50_

^f^The molecular weight (MW) of MA is 365.98 g/mol (PubChem compound database, CID 64953, national center for biotechnology information) (https://pubchem.ncbi.nlm.nih.gov/compound/64953)

^g^Dose employed: 10 mg/mL

Antileishmanial activity was measured by determining the EC_50_ that corresponds to the concentration of drug that gives the half-maximal reduction of the amount of parasites (Table 1). Dose–response relationship showed that only compound **6** was active against intracellular amastigotes of *L.* (V) *panamensis* with an acceptable cytotoxicity (IS: 1.2) when U-937 cells were used as model*.* It is worth mentioning here that in previous studies, compound **6** exhibited an IC_50_ of 69.81 and 26.07 µM on CHO-K1 and SW480 cells lines, respectively, which showed that the cytotoxicity of this compound is not specific against tumor or non-tumor cells (Herrera et al. 2018). Remaining compounds (**3-5** and **7-9**) showed a slight response on intracellular survival and multiplication of amastigotes less than 50% (3.7–32.5%), due to that the anti-leishmanial activity on intracellular amastigotes was not determined. For compound **8** the dose used to determine the inhibition intracellular amastigotes growth was 1.0 µg/ml. It was not possible to increase the concentration due to its high toxicity.

It is important to highlight that only the compounds with two oxygenated positions (methoxy or hydroxy group) in the aromatic ring of the styryl group (**4-7**), showed inhibition over intracellular amastigotes growth.

### Multilevel computational studies

#### Molecular docking studies

Adenine phosphoribosyltransferase (APRT), Trypanothione synthetase (TryS), *N*-myristoyltransferase (NMT), Trypanothione reductase (TryR), Pteridine reductase 1 (PTR1), Tryparedoxin peroxidase (TXNPx), Glyceraldehyde-3-phosphate dehydrogenase (GAPDH), Arginase (ARG), Oligopeptidase B (OPB), Dihydroorotate dehydrogenase (DHODH) have been identified as druggable or potentially druggable targets in Leishmania treatment, which are essential for infectivity, virulence, and viability of insect-stage parasites. In this paper, a multi-target molecular docking protocol were used as a reasonable strategy to suggest a probable mechanism at the molecular level for the most in-vitro active coumarin **6**. To accomplish this goal, we explored the representative contacts from the coumarin structure against the 3D-structures of ten robustly validated drug targets in leishmaniasis drug discovery.

Unfortunately, neither X-ray crystal structures for these proteins have been deposited in the Protein Data Bank archives (PDB), however their protein sequences are successfully studied and deposited in the freely accessible database UniProtKB (https://www.uniprot.org/), therefore, suitable homology models were built for *L. panamensis* by using the bioinformatics web-server SWISS-MODEL (Waterhouse et al. 2018; Bienert et al. 2017; Studer et al. 2020; https://swissmodel.expasy.org/interactive) as follow: model for *Lp*-APRT (obtained from *L. donovani,* PDB: 1QB7, 79.15% identity), model for *Lp-*NMT (obtained from *L. donovani,* PDB: 2WUU, 91.21% identity), model for *Lp-*TryS (obtained from *L. mayor,* PDB: 2VPS, 84.82% identity), model for *Lp*-TXNPx (obtained from *L. mayor,* PDB: 4K1F, 90.03% identity), model for *Lp*-PTR1 (obtained from *L. mayor,* PDB: 5L4N, 77% identity), model for *Lp*-GAPDH (obtained from *L. mexicana,* PDB: 1M66, 84.59% identity), model for *Lp*-OPB (obtained from *L. mayor,* PDB: 2XE4, 85.56% identity), model for *Lp*-ARG (obtained from *L. mexicana,* PDB: 4ITY, 80.56% identity), model for *Lp*-DHODH (obtained from *L. (V.) braziliensis,* PDB: 4WZH, 78.77% identity). Then, in-silico screening using molecular docking was performed to each reported catalytic domain to estimate the protein–ligand binding free energy and docked poses of the active compound **6**. As showed in Table 2, coumarin **6** had larger binding affinity (about − 10.1 kcal mol^−1^) and higher docking-specificities against the eukaryotic enzyme *N*-myristoyltransferase (NMT) in comparison to remaining Leishmania-targets. In addition, note that significant binding score was also predicted when coumarin was docked against the adenine phosphoribosyltransferase enzyme-APRT (− 8.6 kcal mol^−1^). This last finding is particularly interesting and is consistent with previous reports, which have suggested APRT as a promissory drug-target for compounds based on coumarin core (Zaheer et al. 2016).

**Table 2:** Best binding energy (kcal mol^−1^) based on AutoDock vina scoring of the promising coumarin **6** into the active sites of ten key Leishmania protein targets

*Leishmania* target protein	Best binding energy (kcal mol^−1^)	Current inhibitors for NMT	
		*S*-(2-oxo)pentadecylCoa^k^	Myristoyl-CoA^l^
*Lp-*NMT^a^	− 10.1	− 8.9	− 9.2
*Lp-*TryS^b^	− 6.9	–	–
*Lp-*TryR^c^	− 7.3	–	–
*Lp-*TXNPx^d^	− 6.8	–	–
*Lp-*PTR1^e^	− 7.3	–	–
*Lp-*GAPDH^f^	− 7.0	–	–
*Lp-*OPB^g^	− 7.1	–	–
*Lp-*ARG^h^	− 6.3	–	–
*Lp-*DHODH^i^	− 6.9	–	–
*Lp-*APRT^j^	− 8.6	–	–

^a^*Leishmania Panamensis N*-myristoyltransferase

^b^*Leishmania Panamensis* trypanothione synthetase

^c^*Leishmania Panamensis* trypanothione reductase

^d^*Leishmania Panamensis* tryparedoxin peroxidase

^e^*Leishmania Panamensis* pteridine reductase 1

^f^*Leishmania Panamensis* glyceraldehyde-3-phosphate dehydrogenase

^g^*Leishmania Panamensis* oligopeptidase B

^h^*Leishmania Panamensis* arginase

^i^*Leishmania Panamensis* dihydroorotate dehydrogenase

^j^*Leishmania Panamensis* adenine phosphoribosyltransferase

^k^Potent non-hydrolysable inhibitor of NMT protein

^l^Essential cofactor for NMT activity

In this paper, we focus our discussion on the docking results from *N*-myristoyltransferase (NMT), which was estimated as a potential druggable target for the active coumarin **6**. In order to understand the binding behavior of coumarin **6** against NMT enzyme from molecular docking studies, a close view of its possible binding mode was investigated. *N*-myristoyltransferase (NMT), a Leishmania enzyme, is responsible for protein modification through the covalent linkage of the lipid myristate (C14:0) onto the *N*-terminal glycine of specific proteins, which is essential for signal transduction, protein trafficking, and a variety of other cellular functions into the parasite (Corpas-Lopez et al. 2019). To carry out this function, the NMT enzyme uses as essential cofactor myristoyl-CoA to transfer the myristoyl group to parasite target proteins. Thus, different classes of NMT inhibitors are inspired on the structure of myristoyl-CoA, among them *S*-(2-oxo)pentadecyl-CoA has been reported as a potent non-hydrolysable competitive NMT-inhibitor (Paige et al. 1989). In order to provide a comprehensive analysis of the research problem, molecular docking simulations have also been performed using the natural cofactor myristoyl-CoA and the potent inhibitor *S*-(2-oxo)pentadecyl-CoA as positive control to conduct a comparative analysis with the coumarin **6** into the active domain of NMT.

Docking simulations were carried out into the myristoyl-CoA catalytic pocket of *Lp*-NMT enzyme, which comprise both S1 and S2 binding domains. S1 is a hydrophobic region enclosing critical residues, such as Leu169, Phe168, Ile166, Val197, Val192, Ala200, Ile126, Ala127 and Leu208, while S2 corresponds to hydrophilic domain characterized by the presence of Thr203, Met420, Tyr217, Asp83, Gly397, Tyr80, Tyr345, Glu82, Asp84, Tyr326 and Tyr92 (Paige et al. 1989). For blind docking of ligand–protein, a grid box (40 × 44 × 48 points) was centered at the binding site on x = 11.343, y =  − 13.145 and z = 14.869 enclosing all key amino acids required for the enzymatic activity of the NMT protein.

**Fig. 3:**
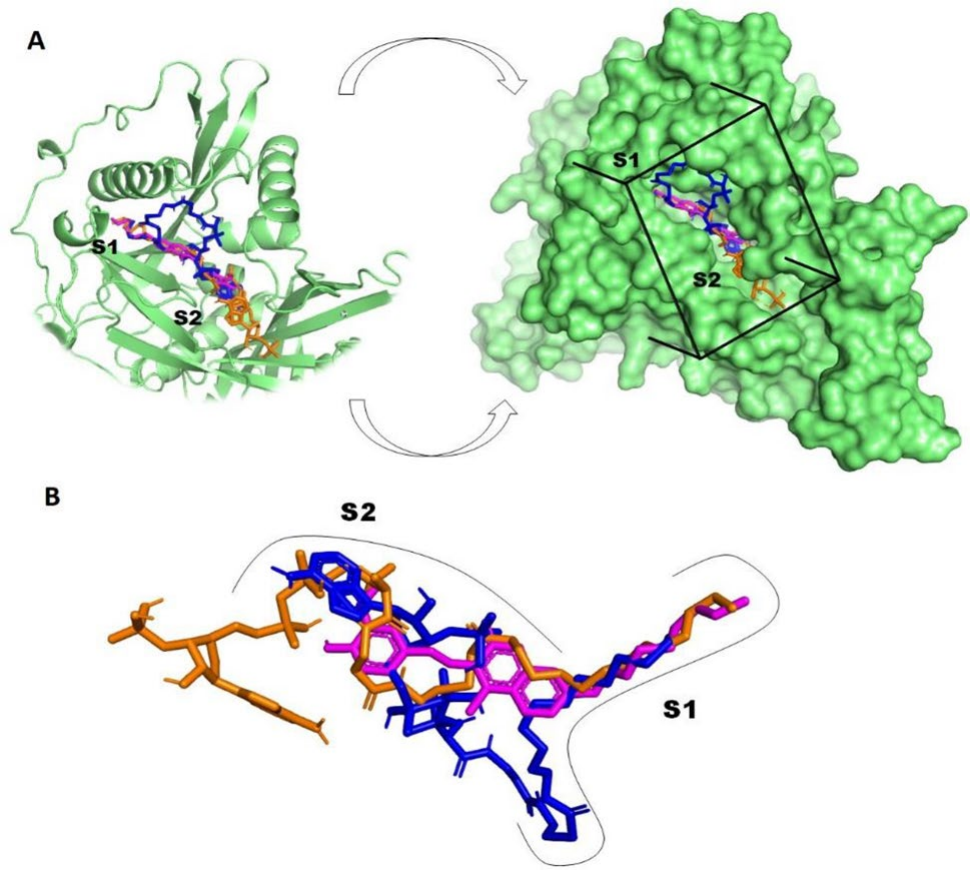
**A** Superimposition of the best binding modes of the coumarin **6** (in magenta) and two known NMT-modulators: myristoyl-CoA (in blue) and S-(2-oxo)pentadecyl-CoA (in orange) alongside Lp-NMT myristoyl-CoA binding pocket. Hydrophobic site (S1) and Hydrophilic site (S2). **B** Superimposed view of the best binding modes for coumarin (in magenta) and myristoyl-CoA (in blue) and S-(2-oxo)pentadecyl-CoA (in orange) (Color figure online)

As mentioned above, docking results showed that coumarin **6** had a better docking score than positive controls (myristoyl-CoA and *S*-(2-oxo)pentadecyl-CoA). This important finding suggests that this compound would have strong binding affinity towards the NMT protein, making it a possible molecular mechanism by which coumarin **6** is capable of interrupting parasite growth. As shown in Fig. 3A, when the most stable binding poses for active coumarin and the inhibitors (myristoyl-CoA and *S*-(2-oxo)pentadecyl-CoA) were plotted into the myristoyl-CoA catalytic domain (the S1 and S2 subsites), we found that coumarin was able to fully accommodate into the myristoyl-CoA catalytic domain on stable conformations during the docking process. Besides, the superimposed analysis indicated similar conformation between the most stable binding pose of **6** and docking NMT—inhibitors complex conformations (Fig. 3B), occupying a location between the S1 and S2 subsites, which suggests that Vina is suitable for docking and screening studies in this work.

**Fig. 4:**
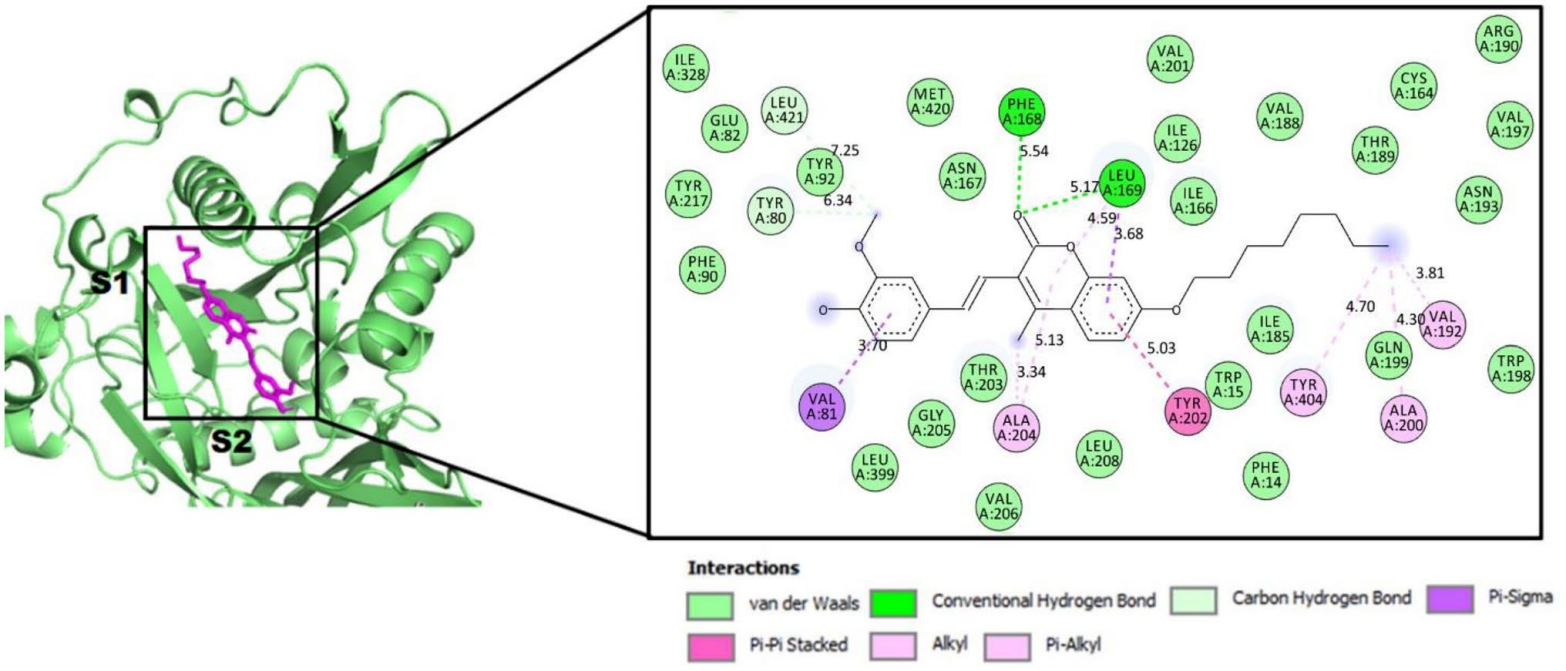
2D ligand–protein interaction plot between coumarin **6** with residues inside the myristoyl-CoA binding pocket of *Lp*-NMT. Dotted lines indicate interactions between coumarin and amino acid residues in the *Lp*-NMT catalytic domain

As current NMT-modulators, coumarin **6** was also able to bind to NMT with at least fourteen amino acids essential for the catalytic activity of NMT as follow: Phe168, Leu169, Ile166, Val197, Val192, Ala200, Ile126, Leu208, Leu169, Phe168 at the S1 domain, as well as Thr203, Met240, Tyr217, Tyr80, Glu82, Tyr92 around the S2 sub-site. This particular result supports our proposal: coumarin **6** might block NMT function with a comparable binding mode to inhibitors preventing the parasite growth. Then, a visual inspection to 2D protein–ligand interaction (Fig. 4) revealed that coumarin **6** displays the occurrence of two moderate strength H-bonds between the carbonyl group on the coumarin motif with key LEU169 (5.17 Å) and PHE168 (5.54 Å) residues, respectively. Various hydrophobic and van der Waals interactions occur in the catalytic site between the active coumarin and twelve residues postulated to bring about the catalytic function of enzyme, such as Ile166, Val197, Val192, Ala200, Ile126, Leu208, Thr203, Met240, Tyr217, Tyr80, Glu82, Tyr92. In addition, we also observed further interactions inside the active pocket of coumarin **6** with NMT that have not been reported yet for current NMT inhibitors as follow: π–π/alkyl contacts with Tyr202, Tyr404 and Val81 and strong hydrophobic interactions surrounded by side chains of Ile328, Phe90, Leu421, Tyr92, Asn167, Val201, Val188, Thr189, Cys164, Asn193, Gln199, Thr203, Gly205, Val206 and Ile185. Both crucial interactions and those additional binding interactions suggesting that the central core and the alkyl long chain is important for inhibiting of *N*-myristoyltransferase (NMT) action.

**Fig. 5:**
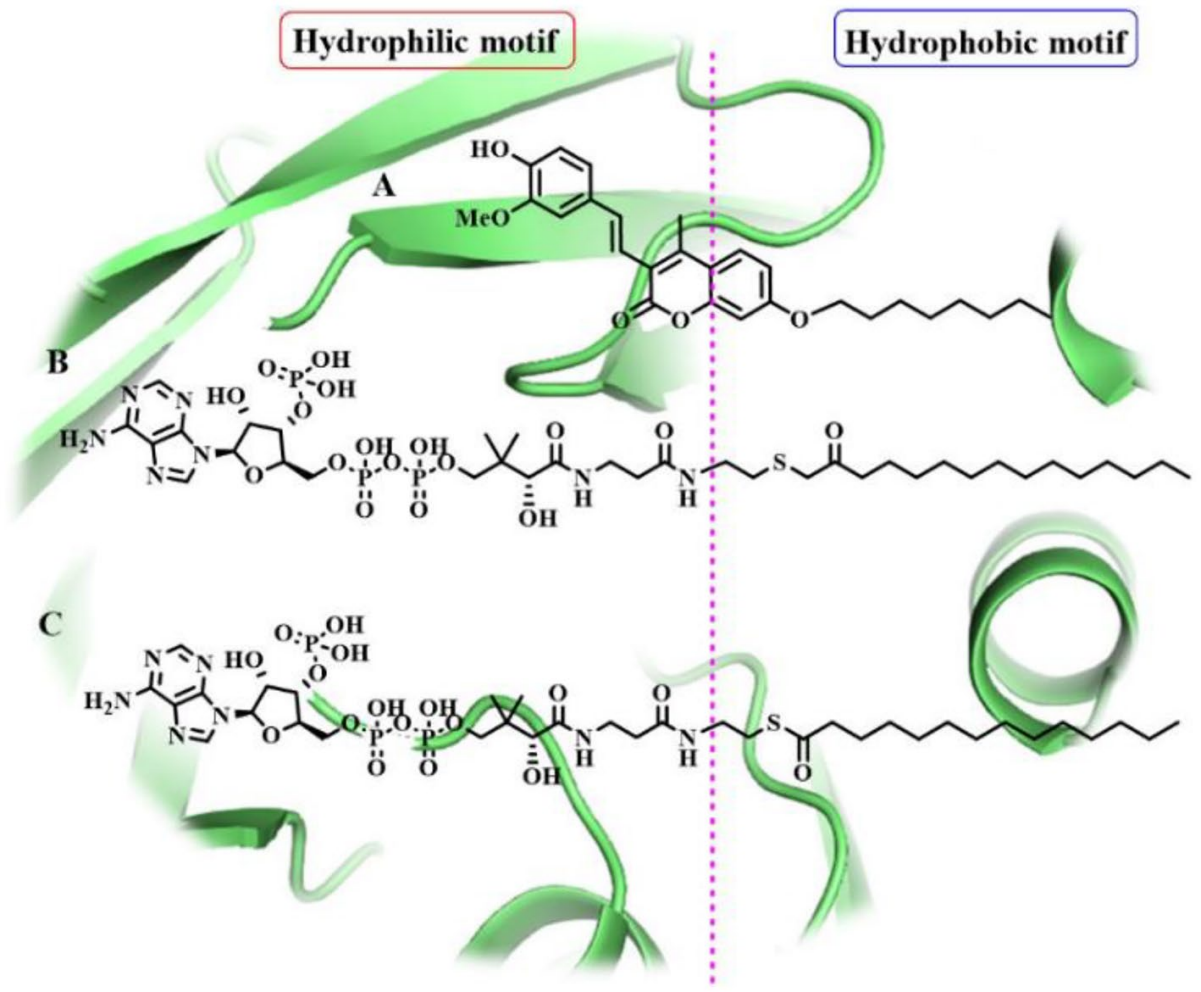
Structural comparison between the active coumarin 6 (**A**) and NMT-inhibitors inhibitors S-(2-oxo)pentadecyl-Coa (**B**) and myristoyl-Coa (**C**). Magenta dotted line separates two possible key structural requirements for NMT-inhibition

Finally, coumarin **6** and NMT-modulators (myristoyl-CoA and *S*-(2-oxo)pentadecyl-CoA) backbones were visually inspected in order to identify the pharmacophoric features that can be used to explain how structurally diverse ligands can bind to a common target, specifically *N*-myristoyltransferase (NMT) enzyme from *L. Panamensis*. It can be seen in Fig. 5, that similar structural hallmarks were found, first a long hydrophobic tail well-conserved in the respective hydrophobic regions of each molecule, followed by a common hydrophilic head moiety. Finding could have important implications for an effective binding to NMT inside the myristoyl-CoA catalytic site and highlight the importance of these pharmacophoric motifs in the future development of selective myristoyl-CoA-competitive inhibitors capable to stall parasite development. It should be noted that the structure of the active coumarin **6** is in good agreement to these key structural requirements, which would suggest that the leishmanicidal response of **6** could be based on the competitive inhibition of *Lp*-NMT enzyme.

#### Molecular dynamics (MD) and post-MM-PBSA studies

**Fig. 6:**
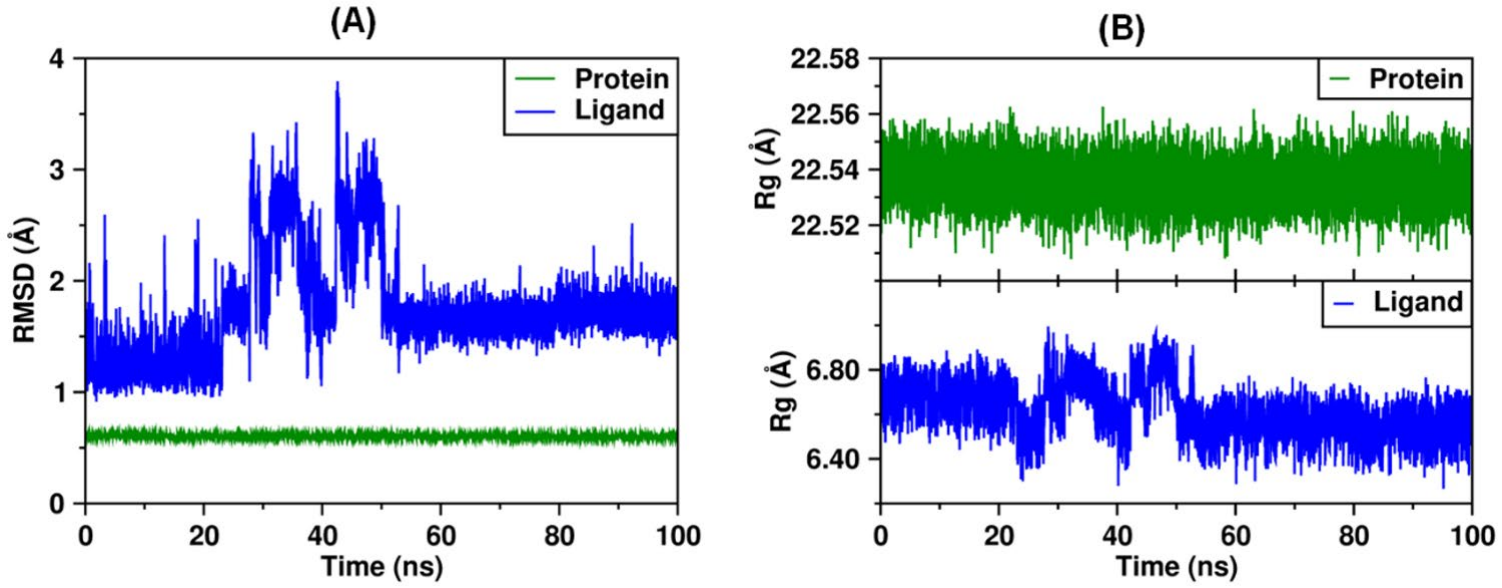
**A** Analysis of RMSD trajectories for the **6**/*Lp*-NMT complex throughout 100 ns all-atom MD simulation. **B** Evolution of the radius of gyration (Rg) for **6**/*Lp*-NMT complex along 100 ns of MDs

To verify the thermodynamic and conformational stability of the best docking solution obtained for the **6**/*Lp*-NMT complex, a 100 ns MD simulation was carried out at physiological conditions (aqueous solution at p = 1 atm, T = 300 K). To this purpose, the root-mean-square deviation (RMSD) after 100 ns MD trajectory with respect to the starting structure was examined. In addition, the absolute binding free energy on **6**/*Lp*-NMT complex was estimated via molecular mechanics Poisson-Boltzmann Surface Area (MM-PBSA) method. As illustrated in Fig. 6A, our findings revealed that coumarin **6** maintained a relatively stable state in the course of the MD simulation with slight changes in backbone structure. A RMSD of the backbone of **6** inside the catalytic pocket on *Lp*-NMT was calculated in about 1.75 ± 0.46 Å, which fall within the optimal range around 2.0 Å (Gohlke et al. 2000; Kramer et al 1999). This result would indicate that **6** was thermodynamically and conformationally stable within the myristoyl-CoA catalytic domain at the course of 100 ns MD simulation period.

Further, the radius of gyration (Rg) was estimated aims at providing more insight regarding **6**/*Lp*-NMT complex stability after the 100 ns MD simulation time. Rg is an index to monitor the stability and the compactness of protein–ligand complexes or proteins during the course of an MD trajectory. Lower values of Rg indicating a more compact structure during simulations. Rg plot for **6** in complex with *Lp*-NMT is illustrated in Fig. 6B.

**Fig. 7:**
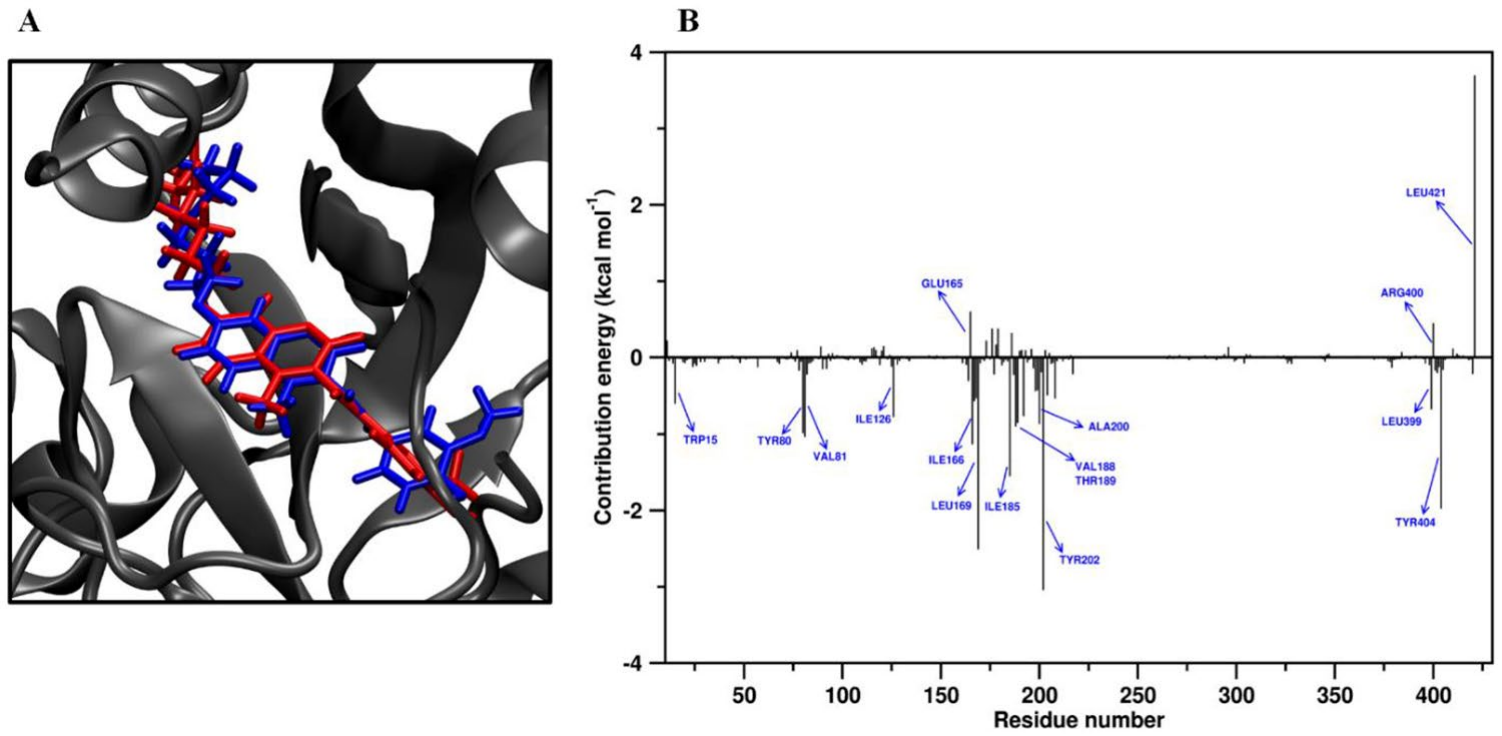
**A** 3D surface plot for comparison the top-scoring docking pose (blue), and the best conformation (in red) of **6** after 100 ns MD simulations inside the myristoyl-CoA catalytic site. **B** The energy contributions per amino acid residue to the **6**/*Lp*-NMT complex formation by MM-PBSA method (Color figure online)

Notably, for **6**, a small radius of gyration was determinate (6.62 ± 0.12 Å), meaning a relatively compact ligand, confirming its stability during the binding occurrence with the *Lp*-NMT protein. These interesting facts support our docking hypothesis, which **6** would affect growth and development of intra-erythrocytic parasites of *L.* (V) *panamensis* via a probable blockage of the *Lp*-NMT function as primary biochemical mechanism. This last statement was also supported by comparing the top-scoring docking pose and the best conformation of **6** in the MD-equilibrated *Lp*-NMT (Fig. 7A), that showed a no dramatic conformational difference between the structure obtained after MD simulation time and the best-docking pose of coumarin **6**.

Considering the above results, we also carried out further analysis by the Molecular Mechanics/Poisson–Boltzmann Surface Area (MM-PBSA) method aims to examine the binding stability of the complex in terms of the different contributions to the free energy upon **6** targeted *Lp*-NMT protein. Decomposed binding free energy of **6** to the *Lp*-NMT protein deposited in Table 3, showed that **6** binds to *Lp*-NMT protein with high affinity (ΔG_bind_ =  − 47.26 ± 0.08 kcal mol^−1^). In particular, breakdown of the binding free energy into its components revealed that van der Waals and electrostatic interactions as well as the non-polar solvation free energy (SASA) positively contributed to the total interaction energy while occurrence of polar solvation are minor favorable contributions to binding. Thereon, the MM-PBSA residue contribution histogram illustrated in Fig. 7B was in good accord with the docking results.

**Table 3:** Binding free energy examination for **6**/*Lp*-NMT complex based on MM-PBSA

Energy contribution	Value (kcal mol^−1^)
ΔG_vdw_^a^	− 63.94 ± 0.08
ΔG_Electr_^b^	− 0.03 ± 0.03
ΔG_Polar_^c^	22.80 ± 0.09
ΔG_SASA_^d^	− 6.09 ± 0.01
ΔG_bind_^e^	− 47.26 ± 0.08

^a^van der Waals contribution to the gibbs energy

^b^Electrostatic contribution to the gibbs energy

^c^Polar solvation energy

^d^Non-polar solvation energy

^e^Total free energy of binding

Thus, amino-acid catalytic residues such as Tyr80, Ile126, Ile166, Leu169, and Ala200 which play an important role in the MMP-7 function, contributing favorably to the global free binding energy profile of the **6**/*Lp*-NMT complex. This fact provides solid evidence to support our mechanistic hypothesis involving **6** targeting *Lp*-NMT. However, aminoacids residues such as Glu165, Arg400, and Leu421 do not favor energetically to the total binding free energy, negatively affecting the interaction of **6** to the probable target protein. The unfavorable contributions to binding of these aminoacid residues appears to result from bad contacts that create steric hindrance, which may contribute to a decrease in the overall binding affinity upon the ligand–receptor binding event.

### Theoretical drug-likeness evaluation of coumarins 4-7

Characteristics such as drug-likeness, pharmacokinetic and physicochemical properties can be used as efficient filters in the development of new anti-parasitic candidates. Early predictions of biopharmaceutical profiling could enhance probability of success in drug discovery settings followed by a significant reduction of time and money, thereby promoting further preclinical and clinical experiments for a lead compound. In this scenario, ten biopharmaceutical parameters were calculated for the coumarins **4**-**7** and then compared against those of 95% of approved drugs (Table 4). As previously discussed, among all the synthesized compounds only coumarin **6** appears to be a new anti-leishmanial scaffold able to inhibit Leishmania *N*-myristoyltransferase (NMT), therefore, in the course of this discussion we would focus only on coumarin **6**. Notably, favorable pharmacokinetics indices were found for compound 10 compared to 95% of FDA-approved drugs. According to the Lipinski rule-five (Lipinski et al. 1997) (violations might be above 1) the tested coumarin **6** could be used as orally administrable drug in humans. Furthermore, **6** was about 100% for human intestinal absorption (% GI), this represents that the coumarin could be absorbed throughout the human gastro-intestinal segments upon oral administration. The degree of lipophilicity (calculated as logP_o/w_) was predicted to be about 5.985, fitting well within the ideal range for lipid-based formulations (–2.0 to 6.0) (Ditzinger et al. 2019) and as was well-correlated with the percent inhibition, which apparently could explain its in vitro behavior.

**Table 4:** Lipinski’s rule and pharmacokinetic score for the synthesized conjugates **4**-**7**

Properties	**4**	**5**	**6**	**7**
MW^a^	450.574	450.574	436.547	450.574
PSA^b^	58.947	59.120	73.994	59.072
n-rot Bond	13	13	13	13
n-ON^c^	5	5	5	5
n-OHNH^d^	0	0	1	0
Log P_o/w_^e^	6.537	6.678	5.985	6.666
LogK_HSA_^f^	1.136	1.151	1.093	1.139
Caco-2 (nm/s)^g^	3124	3081	912	3221
App. MDCK (nm/s)^h^	1694	1669	448	1752
% GI^i^	100	100	100	100
Lipinski’s violations (≤ 1)	1	1	1	1

^a^Molecular weight of the compound (150-500)

^b^ Polar surface area (PSA, Å^2^) (7.0–200)

^c^ n-ON number of hydrogen bond acceptors < 10

^d^ n-OHNH number of hydrogens bonds donors ≤ 5

^e^ Octanol–water partition coefficient (log *P*_*o/w*_) (− 2.0 to 6.5)

^f^ Binding-serum albumin (logK_HSA_) (− 1.5 to 1.2)

^g^ Human intestinal permeation (< 25 poor, > 500 great)

^h^ Madin–Darby canine kidney (MDCK) cells permeation (< 25 poor, > 500 great)

^i^% Human oral gastrointestinal (GI) absorption (< 25% is poor)

In addition, we also calculated the PSA parameter for compound **6**, which correlates passive molecular transport through membranes and drug-membrane interactions (Ertl et al. 2000). Notably, among tested compounds, **6** showed higher PSA value of 73.994 which together with the logP_o/w_ value would appear to be strongly associated to their observed leishmanicidal potency, as will be discussed in more detail later. On the other hand, the in silico passive transmembrane permeation calculated for the title compounds using Caco-2 cell monolayers or MDCK cells as model were also considered. Both models are often recommended as a simplified in vitro model of intestinal absorption after oral administration in drug discovery (Pham-The et al. 2018; Broccatelli et al. 2016; Press and Grandi 2008), thus the most promising compound **6** had 912 and 448 nm/s values of human intestinal permeability, which are well-adjusted within the optimal range for oral formulations approved by FDA. Another approach which predicted the ability of the drug-candidates to bind blood plasma proteins was computed for **6**. Human serum albumin—HSA (calculated as logK_HSA_) is the most critical parameter for distribution and transport of anti-parasitic formulations in the systemic circulation and play an important role in the early stage of drug discovery (Zhivkova 2015; Colmenarejo 2003). Predictive model suggests that compounds having positive numbers should tend to have higher binding affinity to HSA, while negative values could indicate that chemicals show less affinity to HSA binding. Thus, the binding affinity to HSA was calculated to be a positive number of 1.093 for coumarin **6**, which fit well within the recommended range for oral drugs candidates (− 1.5 to 1.5).

**Fig. 8:**
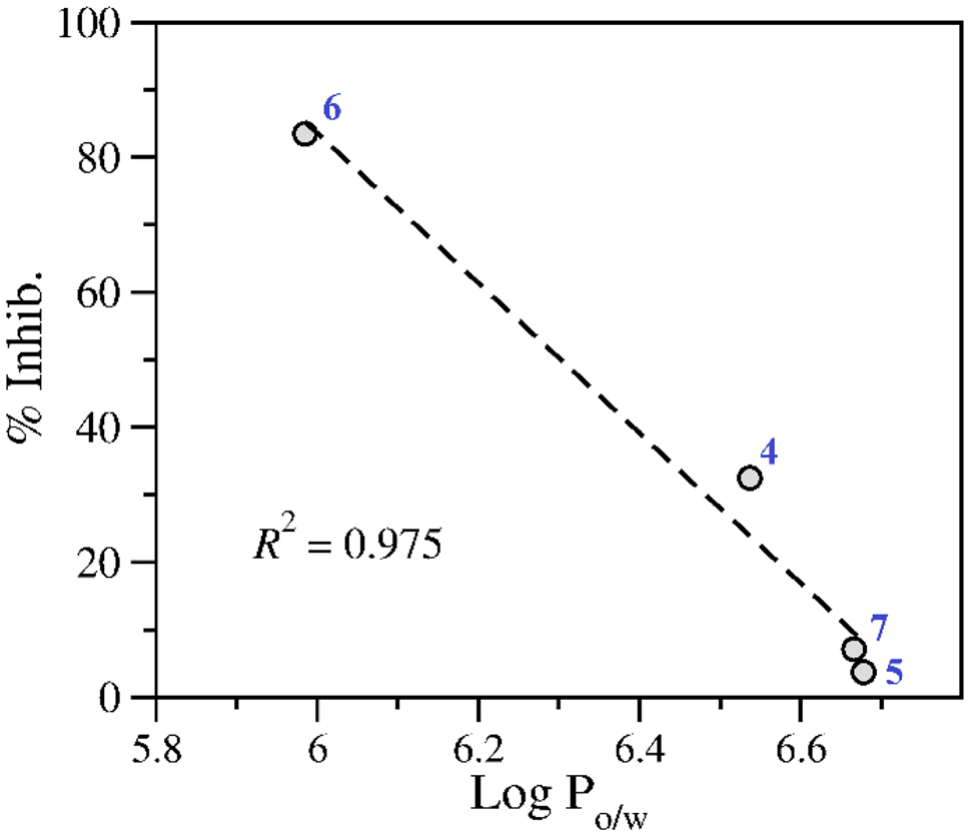
Scatter plot between the predicted logP_o/w_ and the experimental percent inhibition

Finally, pharmacokinetics indices calculations could provide a reasonable explanation as to why coumarin **6** emerged as the most active compound (showing > 83% inhibition of intracellular amastigote growth and EC_50_: 40.5 ± 6.3 µM) from the tested series. Then, a rigorous analysis for these parameters revealed a strong linear correlation between lipophilicity indices (calculated as logP_o/w_) and inhibitory potency when we plot the predicted logP_o/w_ as a function of percent inhibition observed in the in-vitro assay (Fig. 8). It is well-established that lipophilicity is a critical parameter strongly associated to the permeation through the biological cell membranes commonly used to accelerate the development of antiparasitic drug candidates. In this scenario, data from Fig. 8 showed that the inhibitory potency for coumarins **4**-**7** would be apparently conditioned to its lipophilicity index, suggesting may be that among all the tested compounds, coumarin **6** is readily taken up into infected cells in culture, thereafter reducing the number of internalized parasites by an apparent blocking or slowing of the NMT function. This last assumption probably would explain how this compound was able to stimulate a high attenuation of the number of Leishmania intracellular amastigotes (%inhibition: 83.5 ± 5.0 and EC_50_: 40.5 ± 6.3 µM).

From the experimental and computational findings, coumarin **6**, could be a safe drug candidate or its structure might be used as a model for scaffold-based drug discovery in Leishmania chemotherapy. In addition, multi-level virtual results provide strong evidence that the tested coumarin **6** may targeted the protein *N*-myristoylation process into the parasite, which would be responsible for its marked parasite growth inhibition seen in the in vitro assay. However, further enzymatic and preclinical experiments must be carried out to establish the fully leishmanicidal potential for the novel coumarin **6**.

## Conclusion

Antileishmanial, cytotoxic activity and multi-level modelling studies of seven 3-styrylcoumarins are reported. On a structure–activity relationship basis, it is interesting to note that only the compounds with two oxygenated positions (methoxy or hydroxy group) in the aromatic ring of the styryl group (**4-7**), showed inhibition over intracellular amastigotes growth. Among these studied compounds, coumarin **6** reduced the numbers of intracellular parasites with a similar activity than the FDA-approved meglumine antimoniate. This compound was not toxic for mammalian U-937 cells. From modeling studies, compounds were accurately docked directly into the active domain of several druggable Leishmania proteins, which *N*-myristoyltransferase (NMT) enzyme appearing to be a possible mechanism by which more active coumarins, especially the compound **6**, inhibited intracellular parasite growth. Molecular dynamic studies, which have been focused on the coumarin **6**, affirm the docking hypothesis, revealing that the **6**/*Lp*-NMT complex was found to be rather stable during the course of 100-ns MD simulation. Further, post-simulation MM/PBSA protocol have shown that compound **6** binds to the *Lp*-NMT with a total binding free energy (ΔG_bind_) of − 47.26 ± 0.08 kcal mol^−1^, which includes contributions from bonded and nonbonded (nonpolar solvation, electrostatic and van der Waals) interactions to the total interaction energy. Finally, theoretical drug-likeness studies would suggest that the promising** 6** exhibits optimal biopharmaceutical indices to be considered in further pre-clinical testing. Taken altogether, coumarin **6** of the present study could serve as a lead in the future for development of more effective leishmanicidal agents. However, further theoretical and experimental assays are needed to further validate these preliminary findings.
